# Dual Carbonaceous Materials Synergetic Protection Silicon as a High-Performance Free-Standing Anode for Lithium-Ion Battery

**DOI:** 10.3390/nano9040650

**Published:** 2019-04-23

**Authors:** Xing Li, Yongshun Bai, Mingshan Wang, Guoliang Wang, Yan Ma, Yun Huang, Jianming Zheng

**Affiliations:** 1 The Center of New Energy Materials and Technology, School of Materials Science and Engineering, Southwest Petroleum University, Chengdu 610500, Sichuan, China; bysdyr@163.com (Y.B.); wangguoliang1012@163.com (G.W.); mayanlouis@163.com (Y.M.); huangyun982@163.com (Y.H.); 2 Research Institute (RI), NingDe Amperex Technology Limited, Ningde 352100, Fujian, China

**Keywords:** silicon, carbon coating, reduced graphene oxide, self-standing film, anode

## Abstract

Silicon is the one of the most promising anode material alternatives for next-generation lithium-ion batteries. However, the low electronic conductivity, unstable formation of solid electrolyte interphase, and the extremely high volume expansion (up to 300%) which results in pulverization of Si and rapid fading of its capacity have been identified as primary reasons for hindering its application. In this work, we put forward to introduce dual carbonaceous materials synergetic protection to overcome the drawbacks of the silicon anode. The silicon nanoparticle was coated by pyrolysed carbon, and meanwhile anchored on the surface of reduced graphene oxide, to form a self-standing film composite (C@Si/rGO). The C@Si/rGO film electrode displays high flexibility and an ordered porous structure, which could not only buffer the Si nanoparticle expansion during lithiation/delithiation processes, but also provides the channels for fast electron transfer and lithium ion transport. Therefore, the self-standing C@Si/rGO film electrode shows a high reversible capacity of 1002 mAh g^−1^ over 100 cycles and exhibits much better rate capability, validating it as a promising anode for constructing high performance lithium-ion batteries.

## 1. Introduction

Rechargeable lithium-ion batteries (LIBs) have been successfully used in daily applications during the past decades due to their high energy density, lightweight, and environmentally friendly nature [1,2,3,4,5,6,7]. In the burgeoning market of electric vehicles, there is a growing demand for developing rechargeable LIBs with higher energy density, higher safety and longer life cycle [8,9]. However, LIBs using the currently commercialized materials can only achieve about ~250 Wh kg^−1^ [10], which is unsatisfactory compared with a vehicle using internal combustion engines. Thus, many efforts have been made in search of new cathode/anode electrode materials to construct LIBs with higher energy/power densities [11,12,13].

Recently, silicon-based materials have attracted much attention because of their highest theoretical specific capacity of 4200 mAh g^−1^ and low discharge potential (~0.4 V vs. Li/Li^+^), abundant resources, etc., which is a promising candidate to replace graphite anode for LIBs [11,14,15,16]. However, the extremely high volume expansion (up to 300%) upon the lithium ion insertion process and unstable formation of solid electrolyte interphase (SEI) film due to the repeated insertion/extraction of lithium ions, result in the pulverization of particle and rapid fading of capacity [17,18,19,20,21].

To address the above issues of the silicon anode, the introduction of a second phase (e.g., carbon) is the one popular tactic to solve the particle pulverization, because the carbon matrix can suppress large lithiation/de-lithiation-induced strains and fracture [12,17,22,23]. On the other hand, the carbon second phase can also function as a protective barrier to prevent direct exposure of silicon in the electrolyte and thus enhance the interface stability of the electrode. Accordingly, various research works have been recently reported on Si-based anodes, such as core-shell Si@C composites [24], hollow Si/C nanoparticle [25,26,27], Si/C nanofibers [28,29,30,31,32], Si/Graphene nanosheets [33,34,35,36,37], etc. Some fabrication methods are facile and can realize improvements for the electrochemical performance of Si anode. There are still some problems existing with a single carbon specie to fabricate Si-based composites, such as particle agglomeration, poor conductivity, slow fracture of the matrix, and the unstable interface between Si and electrolyte upon long-term cycling [13]. Thus, it is important to introduce dual carbonaceous materials to maintain good electrical connections by designing a continuous conductive network along with conformal coverage of a large range. It is critical to retain stable active materials surface/interface by coating with a protecting carbon layer on the surface of the Si to avoid parasitic side reactions with the electrolyte [38,39]. Also, introduction a secondary carbon phase can also further provide good electrical connections between active particles among the entire electrode. As a result, the dual carbonaceous materials introduced in Si-based materials usually exhibit better cycling stability and rate performance. Some classic dual carbonaceous-Si composites materials include: carbon coated Si@graphene [11], Si/CNT/grapheme [40], Si/carbon nanofiber/carbon [41,42] etc.

Recently, the development of flexible and lightweight electrode for lithium ion battery has been considered a vigorous technology for the next-generation electronics devices, such as wearable devices and smart electronics. Fabricating flexible self-standing electrodes without binder and conductive agent is one of the important methods to enhance the gravimetric capacity of practical electrodes for a lithium ion battery. Much research has been focused on the fabrication of self-standing electrodes for lithium ion battery, such as self-standing Li_4_Ti_5_O_12_/carbon [43], metal oxide-carbon [44,45], metal sulfide-carbon electrode [46,47]. Fabrication of Si based self-standing electrode has also been developed using carbon nanofiber [48], grapheme [49,50], carbon cloth [51,52,53] as carbon matrix. Although there are a lot of self-standing Si-based materials for the lithium ion battery, it is still a great challenge to fabricate flexible electrode with high electrochemical stability. This is especially the case for silicon, whose low conductivity strictly limit its practical capacity without conducive agent. Also, the huge volume changes during the repeated cycling process may reduce its cycling stability without binder.

Inspired by the pervious work, we put forward a way to introduce dual carbonaceous materials to fabricate a self-standing carbon coated silicon/reduced graphene oxide (C@Si/rGO) film to synergistically protect the silicon as a high-performance anode material. The freestanding C@Si/rGO film anode is fabricated by using a method of extraction and filtration at room temperature. Poly diallyl dimethy lammonium chloride (PDDA) was used as a coating carbon precursor to charging the Si particle with electropositive properties [1,8,9]. Then, elastic graphene networks were introduced by dispersing graphene oxide solution and self-assembly of the Si@PDDA nanoparticle with electrostatic interaction forces. After freeze-drying and pyrolysis, the PDDA was converted to an inner amorphous carbon layer and tightly anchored the Si particle on the graphene sheets. The architecture of C@Si/rGO film possesses a three-dimensional porous structure, which can remain flexibility and mechanical stability during charge/discharge. In addition, the amorphous carbon can avoid direct contact between silicon particles and electrolyte, further improving the stability of interface Si by formation of a stable SEI layer. In the meantime, the two-dimensional graphene sheets interconnect the isolated Si@C particle, constructing a better continuous electron transportation network within the electrode. Moreover, the dual carbonaceous framework structure constructs a self-standing conductive framework and realizes the flexibility of the electrode, which is free of current collector, conductive agent and binder. The self-standing C@Si/rGO film electrode delivered a high reversible capacity of 1002 mAhg^−1^ over 100 cycles and exhibited much better rate capability than self-standing silicon/reduced graphene oxide (Si/rGO, 740 mAhg^−1^ at 2 Ag^−1^) for LIBs.

## 2. Materials and Methods

### 2.1. Materials

Silicon nanoparticles (size: 20~50 nm) were purchased from Xuzhou Jiechuang New Material Technology Co., Ltd. Poly dimethyl diallyl ammonium chloride(PDDA, 20 wt%) was supplied by Shanghai Aladdin Bio-Chem Technology Co. Ltd. (Shanghai, China). Sulfuric acid (H_2_SO_4_, 95.0~98.0%), Hydrogen peroxide (H_2_O_2_, 30%) and sodium hydroxide (NaOH, ≥98.0%) were provided by Chengdu Kelong Chemical Reagent Factory (Chengdu, China). Graphene oxide (GO, ≥99.0 wt%) was obtained from Da Ying Ju Neng Technology Development Co., Ltd. (Suining, China).

### 2.2. Synthesis of Poly Diallyl Dimethy Lammonium Chlorid -Coated Si (PDDA@Si)

Typically, the graphene oxide solution (GO) was prepared by dispersion of 0.2 g graphene oxide powders in 100 mL deionized (DI) water under repeated ultrasonic dispersion and stirring overnight, which finally formed a homogeneous suspension. Then a piranha solution was prepared by mixing 15 mL of concentrated sulfuric acid (H_2_SO_4_) and 5 mL of hydrogen peroxide (H_2_O_2_) by stirring. After that, 0.1 g silicon (Si) were added into the piranha solution and stirred for 6 h at 80 °C. Next, the mixture was filtered and washed with a large amount of DI water to remove residual H_2_SO_4_ and H_2_O_2_, and then placed it into a vacuum oven at 60 °C for 24 h. Then, the dried Si nanoparticle were dispersed in the solution which was mixed of 200 mL DI water and 2 mL PDDA solution with ultrasonic stirring for 24 h. The excess PDDA solution was removed by repeated washing using deionized (DI) water. Finally, the PDDA-coated Si (PDDA@Si) particle were obtained by drying at 60 °C for 24 h.

### 2.3. Synthesis of Carbon Coated Si/rGO Composite Film (C@Si/rGO) by Electrostatic Self-Assembly

The pH value of the graphene oxide solution was firstly adjusted to 5 by using sodium hydroxide solution with 2 mg/mL. And then, 40 mL of 2 mg/mL of the GO solution was ultrasonic dispersion for 2 h. Next, 13.3 mg PDDA@Si were added to the above GO solution to form a homogeneous mixture and stirred for 24 h by electrostatic self-assembly. After that, the homogeneous mixture solution was filtered by using a 0.2 μm thickness porous polycarbonate (PC) membrane (47 mm in diameter) to prepare the PDDA@Si/rGO self-standing films. Finally, the self-standing C@Si/rGO film with the thickness about 55 μm was obtained through further freeze-drying for 12 h and heat treatment at 500 °C for 5 h within an argon atmosphere. For comparison, the Si/rGO film with the similar thickness was also prepared with the same procedure without treating silicon with PDDA.

### 2.4. Characterization

The zeta potentials of the PDDA@Si and GO solution were measured on a Zeta PALS (American Brookhaven) at room temperature. X-ray powder diffraction (XRD) measurements were measured with an XPERT-PRO diffractometer using Cu Kα radiation (λ = 0.15406 nm) with the 2θ range from 10° to 80°. Thermogravimetric analysis (TGA) was measured with a STA449F3 analyzer (Netzsch, Germany) at air atmosphere with a ramp rate of 10 °C min^−1^. The N_2_ absorption/desorption isotherms associated with specific surface area and pore diameter distribution data were performed on a V-Sorb 2800 P analyzer under 77 K. Raman spectroscopy analysis was measured by using the Renishaw (RM 1000-Invia) with the laser (λ = 785 nm) in a wavenumber range of 100–3500 cm^−1^. Scanning electron microscopy (SEM) was carried out with a FEIINSPECT-Fat with 20 KV accelerating voltage. High-resolution transmission electron microscopy (HRTEM) was acquired on a Libra 200 FE.

### 2.5. Electrochemical Tests

The C@Si/rGO film was cut into disc with the diameter of 14 mm and used as the electrode. The mass of the electrode included the whole wafer weight, including the weight of Si, C and rGO, which is about 1.8 mg cm^−2^. The thickness of the electrode was about 55 μm. The counter electrode and separator were lithium foil (thickness: 250 μm) and polypropylene (PP), respectively. The electrolyte was 1 M LiPF_6_ in ethylene carbonate (EC)/diethyl carbonate (DEC) (1:1 in volume) with 5 wt% fluoroethylene carbonate (FEC) additive. The coin cell (CR2032) consisting of the C@Si/rGO self-standing film anode and Li metal anode was assembled in an Ar-filled glove box with the H_2_O and O_2_ contents less than 0.1 ppm. The CR2032 coin cell was tested using the BTS-5V 20 mA battery testing system (NEWARE Electronic Co. Ltd., Shenzhen, China) in 0.01–1.5 V cut-off voltages. The cyclic voltammetry (CV) with the voltage window 0.01–1.5 V and electrochemical impedance spectroscopy (EIS) over the frequencies in the range from 100 kHz to 100 mHz were recorded by a CHI 660C electrochemical work-station.

## 3. Results

**Figure 1:**
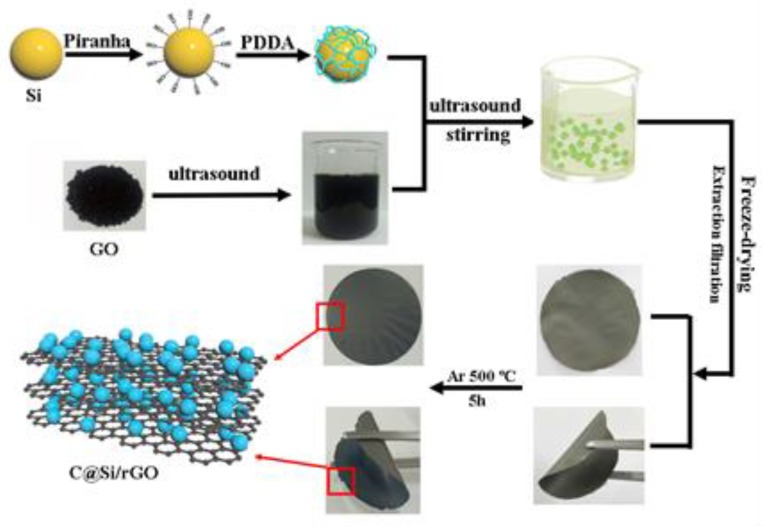
Schematic illustration for preparation of C@Si/rGO self-standing film.

The fabrication process is illustrated in Figure 1. Firstly, the Si nanoparticles were modified by piranha solution, which increase the hydrophilic interaction of of Si by the function of H_2_SO_4_ and H_2_O_2_ [12]. Then, the -NH_2_ group of PDDA could be easily grafted on the hydroxylated Si and coated on the surface of Si with positive charges. On the other hand, GO solution was prepared by ultrasonic dispersion and the pH value was adjusted at 5 by introduction of -OH groups with negative charges. Then, the PDDA@Si was directly mixed with the GO solution to realize electrostatic self-assembly process. Subsequently, a self-standing PDDA@Si/GO film was obtained by filtering the above solution followed by freeze-drying for 12 h to preserve the structure of composites. Finally, the self-standing C@Si/rGO film could be obtained by heat treatment at 500 °C for 5 h in argon atmosphere. As shown in Figure 1, the flexibility of the film can be well retained despite bending.

Figure S1 presents the SEM image and corresponding Energy Dispersive Spectrometer (EDS) mapping of the as prepared PDDA@Si sample. The uniformly dispersed C, N and Cl elements contained in the PDDA are found in the EDS mapping, which indicates the PDDA has been well covered on Si nanoparticle. Moreover, the O element is also found in the EDS mapping, which suggests that the Si nanoparticle has been well modified by piranha solution. Figure S2 shows the Fourier transform infrared (FT–IR) spectra of the PDDA@Si sample. There are two characteristic peaks located at 3465 and 1100 cm^−1^, respectively, which correspond to the vibration of O–H and Si–O bonds of Si nanoparticle [54], further indicating the Si nanoparticle has been well modified by the piranha solution. By comparing the FT–IR spectra of Si and KBr, two peaks were found located at 1640 cm^−1^ and 1181 cm^−1^, respectively, which are assigned to the KBr and C-N of PDDA, [55] also further proving that there are PDDA on the surface of the Si nanoparticle.

**Figure 2:**
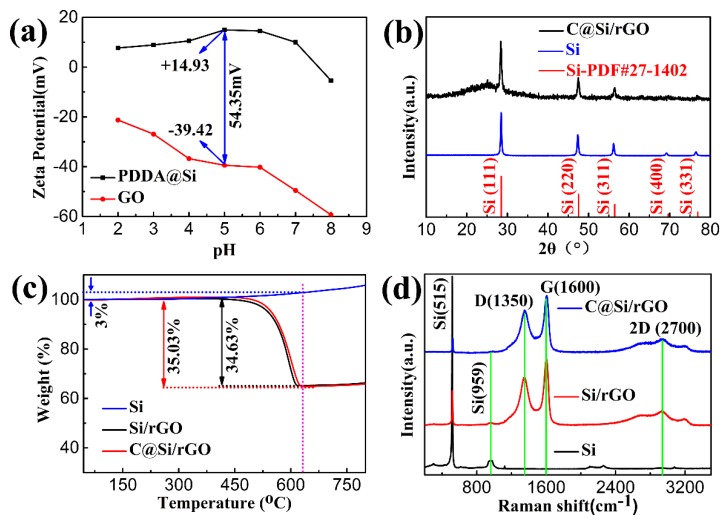
(**a**) Zeta potential of GO and PDDA@Si solution under different pH values. (**b**) XRD patterns of Si nanoparticle and C@Si/rGO film. (**c**) TGA curves of Si/rGO and C@Si/rGO film. (**d**) Raman spectroscopies of Si nanoparticle, Si/rGO film, and C@Si/rGO film.

Figure 2 presents the Zeta potential of GO and PDDA@Si at different pH values. When the Si particle are modified by PDDA, the zeta potential values of PDDA@Si gradually increase from negative values to positive values, up to 14.93 mV at pH value of 5. Meanwhile, the zeta potential values of GO solution were maintained at negative values at pH value of 2–8. When adjusting the pH at 5, there is a maximum absolute value up to 54.35 mV, which means the PDDA@Si and GO is easiest for realizing electrostatic self-assembly. Figure 2b illustrates the XRD patterns of the C@Si/rGO composite and pure Si nanoparticles. By comparing with Joint Committee on Powder Diffraction Standards (JCPDS) card of Si (No. 27–1402), it could be observed that all the peaks can be well indexed to the standard peaks of silicon. The peaks located at 28.6°, 47.5°, 56.3°, 68.5° and 75.7° are corresponding to the (111), (220), (311), (400) and (331) crystal planes of Si, respectively. In addition, there is a significant wide peak located at 20–25° that could be as assigned to the (002) peak of multilayer graphene by comparing with the peaks of pure Si nanoparticles [12]. Figure 2c presents the TGA patterns of the Si/rGO and C@Si/rGO films, respectively. The main weight loss could be clearly seen from 450 °C to 650 °C, which is attributed to the decomposition of carbon. There was an increase in the weight between 650 °C and 800 °C, corresponding to oxidation of Si to SiO_2_ under air atmosphere. Thus, the quantity loss for Si/rGO and C@Si/rGO can be calculated from Equations (1) and (2) [56].

X_1_ + Y_1_ = 1; 1.03X_1_/(1.03X_1_ + 0.3340) = 1 − 0.3340 (1)

X_2_ + Y_2_ = 1; 1.03X_2_/(1.03X_1_ + 0.3337) = 1 − 0.3337 (2)

where, X_1_ and X_2_ are the content of silicon in Si/(C + rGO) and Si/C, respectively. Y_1_ and Y_2_ are the content of carbon in Si/(C + rGO) and Si/C, respectively. The detailed calculation process is described as follows. The weight percentage of Si nanoparticles, which were heated from 40 °C to 800 °C with a heating rate of 10 °C/min, increased to 103% at 650 °C. Likewise, the Si nanoparticle in C@Si/rGO and Si/rGO film will also increase by 3% at 650 °C. In C@Si/rGO and Si/rGO film, we assume the weight percentage of Si/(C + rGO) and Si/C is X_1_/Y_1_ and X_2_/Y_2_, respectively. According to the data from the TGA results (Figure 2c), also based on Equations (1) and (2), we could calculate the values of X_1_/Y_1_ and X_2_/Y_2_. These results suggest that the weight percentage of C + rGO in the C@Si/rGO film is 37 wt% and the rGO in the Si/rGO film is 36 wt%.

In order to further investigate the degree of disorder of the samples, the Raman spectra of Si, C@Si/rGO and Si/rGO film anode were performed and the results are shown in Figure 2d. There are two peaks located at 515 cm^−1^ and 959 cm^−1^ for Si, corresponding to the typical Raman mode of crystalline Si. The Raman spectra of the C@Si/rGO and Si/rGO exhibits two characteristic peaks at 1350 cm^−1^ and 1600 cm^−1^, respectively. The D band is specifically for A1g breathing by sp3 carbon, whereas the G band is the in-plane stretching of sp2 carbon, respectively [20,21]. In addition, the peak locked at 2700 cm^−1^ imply that the exists of rGO in the two samples. The intensity ratio of I_D_/I_G_ was 0.76 for C@Si/rGO anode, which is higher than 0.74 for Si/rGO, indicating the higher disordered property of C@Si/rGO, which is considered to have resulted from the amorphous carbon by the carbonization of PDDA at low temperature [21].

**Figure 3:**
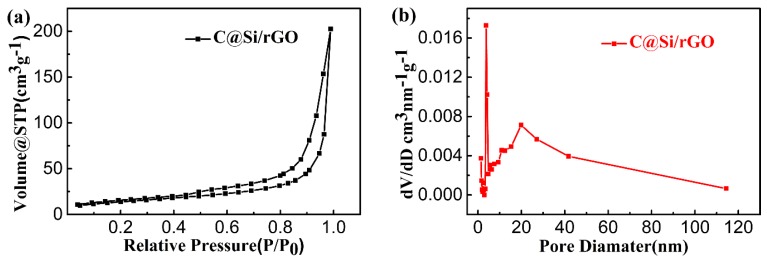
(**a**) N_2_ isotherm absorption-desorption curves of C@Si/rGO film; (**b**) Pore-size distribution of C@Si/rGO film.

Figure 3a,b show the N_2_ adsorption/desorption isotherms curve and pore size distribution curve of the C@Si/rGO film. The C@Si/rGO film displays the fourth type (IV) of adsorption/desorption isotherm curve and H3 type hysteresis loop, implying that there is a layered porous structure caused by multi-layer adsorption of mesoporous materials [57,58]. From the pore size distribution (Figure 3b), this presents an intensive peak at ~3.8 nm, which might be caused by small pores in the overlap of the multi-graphene layers. In addition, a broad peak of ~20 nm can also be found in Figure 3b, which might be attributed to large voids formed by the crosslinking of the graphene framework after freeze-drying [59,60]. According to the tested results as shown in Table 1, the specific surface area, pore volume and average pore diameter of C@Si/rGO material are calculated to be 50.00 m^2^/g, 0.32 cm^3^/g and 25.10 nm, respectively. The porous microstructure are favorable to Li^+^ diffusion to the surface of active materials by shortening the transport pathway.

**Figure 4:**
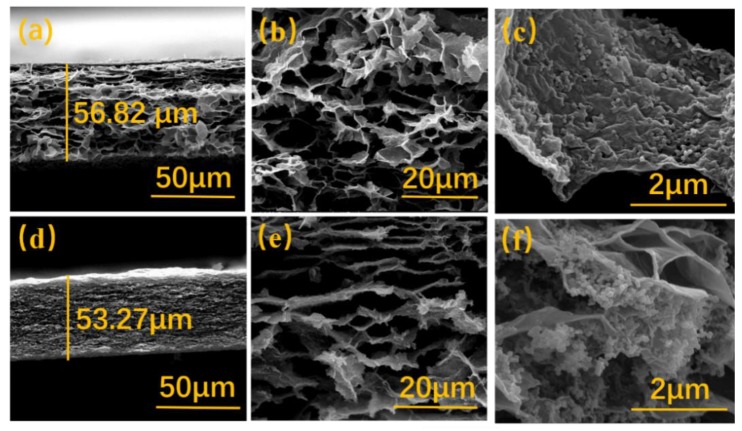
(**a**–**c**) Cross-sectional scanning electron microscope (SEM) images of C@Si/rGO film, and (**d**–**f**) Si/rGO film under different scanning magnification.

The cross-section SEM images of Si/rGO and C@Si/rGO film under different scanning magnification are shown in Figure 4. According to the Figure 4a,d, it could be clearly seen that two-dimensional graphene sheets are layer by layer stacking after vacuum filtration, which represents a good self-standing structure. In addition, the thickness of C@Si/rGO and Si/rGO films is both around 60 μm, which is similar to other reported self-standing electrodes [12]. From Figure 4b,e, abundant three-dimensional voids can be observed as a result of the cross linking between graphene layer. The void structure for C@Si/rGO is significantly more ordered than that of Si/rGO. The three-dimensional porous structure not only provides a buffer for the entire electrode expansion/shrinkage, but also provides a fast electron/ion transport channels. Furthermore, by zooming in on the SEM Si/rGO (Figure 4f) film, it is found that a large amount of Si nanoparticle are easily agglomerated. Moreover, some Si nanoparticle are even not connected with the rGO nanosheets, which might seriously reduce the electrochemical activity of the electrode. In contrast, it could be clearly observed that the Si nanoparticles are uniformly distributed and anchored on the surface of the rGO nanosheets for the C@Si/rGO film (Figure 4c). The uniform dispersion and tight connection between Si nanoparticls and rGO nanosheets secure excellent electrical connections between active material and matrix during the charge/discharge process. Besides, the Si nanoparticle are wrapped by wrinkled graphene layers without being directly exposed to the electrolyte, which is favorable to buffer the volume change during the lithium ion intercalation/extraction and suppress deleterious side reactions.

**Figure 5:**
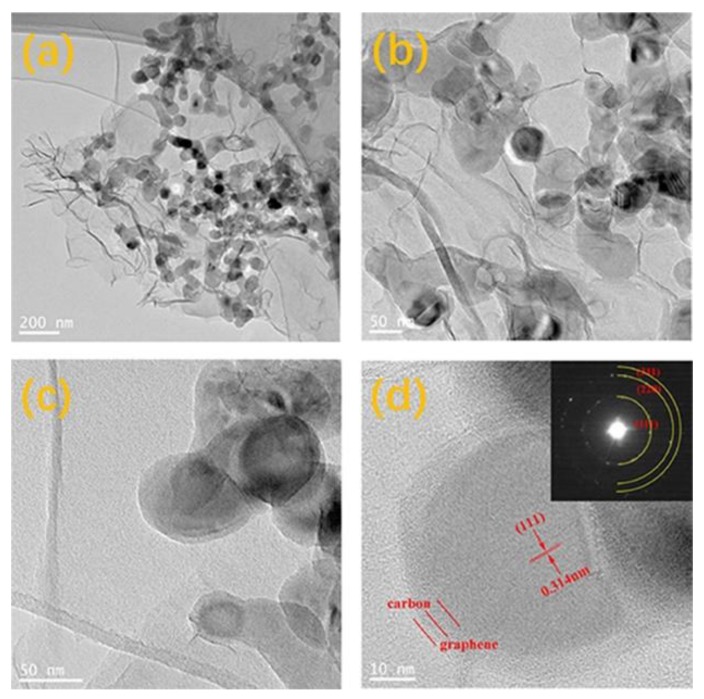
(**a**–**d**) Transmission electron microscope (TEM) images of C@Si/rGO film under different magnification and the selected electron diffraction (SEAD) pattern of Si as inserted in Figure 5d.

In order to further testify the tight junction between Si nanoparticle and graphene nanosheets, the TEM of C@Si/rGO was performed and the result is presented in Figure 5. As shown in Figure 5a,b, the Si particle with ~50 nm size are distributed among the rGO layers without significant agglomeration. There are many brightly white spots near the edge of Si nanoparticle, which should be due to the porous structure from stacking of rGO layers. The result is consistent with the N_2_ gas adsorption/desorption isotherms curve and pore-size distribution curve analysis conclusion [58]. In the HRTEM of Figure 5c,d, the wrinkled edge confirms that the rGO in the C@Si/rGO film is multilayer. The selected electron diffraction (SEAD) pattern as inserted in Figure 5d shows that there are three diffraction rings, which correspond to the (111), (220) and (311) crystalline planes of the Si nanoparticle. Moreover, it also could be observed that the Si nanoparticle is uniformly coated by an amorphous carbon layer in Figure 5d, which should have come from the pyrolysis of the PDDA. In addition, Figure 5d also exhibits that the coating carbon layer could make the Si nanoparticle tightly anchored on the surface of the rGO nanosheet. These results mean that the Si nanoparticle are protected by the two carbonaceous materials of pyrolysis carbon and rGO. The coated pyrolysis carbon layer could protect the Si nanoparticle from being directly exposed to the electrolyte, which is favorable for suppressing an undesired side reaction. Furthermore, as the carbon layer could tightly connect the Si nanoparticle to the flexible and conductive rGO nanosheets, it is also fruitful to improve the surface electronic conductivity and buffer the volume expansion of the Si nanoparticle during the electrochemical process.

**Figure 6:**
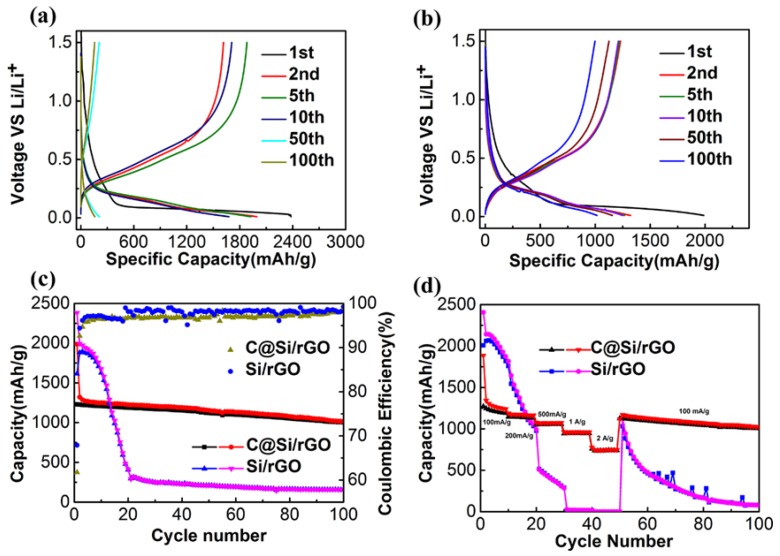
(**a**,**b**) Galvanostatic discharge/charge curves of Si/rGO and C@Si/rGO film electrodes; (**c**) long-term cycling performances of Si/rGO and C@Si/rGO film electrodes at current density of 100 mAg^−1^; (**d**) rate capabilites of Si/rGO and C@Si/rGO film electrodes under different current rates.

Figure 6a,b are the galvanostatic charge/discharge profiles of Si/rGO and C@Si/rGO film electrodes at the 1st, 2nd, 5th, 10th, 50th and 100th cycle, respectively, tested under the current density of 100 mAg^−1^ between 0.01 and 1.5 V vs. Li/Li^+^. It is obvious that the initial discharge cure displays two plateaus at 1–1.2 V and below 0.2 V for Si/rGO and C@Si/rGO, corresponding to the formation of a SEI film and the lithiation of crystalline Si nanoparticle, respectively, which is in agreement with the CV results shown as follow. By comparing the galvanostatic charge/discharge profiles of Si/rGO and C@Si/rGO anode at the 1st, 2nd and 5th cycle, it is found that the polarization of Si/rGO anode shows an increased tendency while which is absent in the C@Si/rGO anode. The polarization of Si/rGO anode increases dramatically and the specific capacity shows a sharp drop at the 10th, 50th and 100th. In contrast, the increase of polarization of the C@Si/rGO anode is negligible and the specific capacity decay is limited. For the Si/rGO anode, due to the agglomeration of Si particle and the large volume expansion and contraction in the process of lithation/delithiation, the active materials cannot maintain good electrical contact with the rGO, leading to a sharp decline of specific capacity. However, for the C@Si/rGO anode, the silicon particles are dispersed uniformly and completely encapsulated in graphene and the silicon particle surface with an additional protective amorphous carbon layer, so the volume expansion and shrinkage are well buffered. Good electrical contact between active substances and the Si/electrolyte interface can be better maintained within the electrode. Therefore, the C@Si/rGO anode exhibits minor polarization and slower specific capacity decay. Figure 6c shows the long-term cycling performances of the Si/rGO and C@Si/rGO anodes under the current density of 100 mAg^−1^ with the voltage between 0.01 and 1.5 V. It could be intuitively found that the cycling stability of the C@Si/rGO anode is much better than Si/rGO anode. The initial charge/discharge capacities of the Si/rGO film anode are 1615 mAh g^−1^ and 2380 mAh g^−1^, respectively, with an initial Coulombic efficiency of 67.9%. However, the following charge/discharge capacity dramatically decreases, and remains at a charge capacity of only 210 mAh g^−1^ after 50 cycles. The sharp capacity loss are attributed to the agglomeration of Si particle, the poorly preserved electrical contact between active materials and rGO, and the pulverization of active substance owing to the large volume variation in the process of charging/discharging. However, the C@Si/rGO anode exhibited the charging/discharge capacity of 1229/1988 mAh g^−1^ in the initial cycle with the Coulombic efficiency of 61.8%. After 50 and 100 cycles, the C@Si/rGO electrode still remain the capacity of 1122/1155 mAh g^−1^ and 1015/1002 mAh g^−1^, respectively, which is much higher than those of Si/rGO film electrode (210 mAh g^−1^ and 157 mAh g^−1^ after 50 and 100 cycles). The better cycling stability is related to the porous structure of C@Si/rGO. In the SEM of C@Si/rGO (Figure 4a,b), the interconnected graphene nanosheets construct abundant macopores, and this pore structure increases the flexibility of electrode and give the Si efficient void to suppress volume change during cycling. Furthermore, the mesoporous structure (Figure 3b and Figure 5b) originates from the stacking and wrinkling of graphene nanosheet provide lots of channels to facilitate Li+ diffusion during the electrode, finally guarantee entire conductive framework structure. Figure 6d shows the rate capability of the Si/rGO and C@Si/rGO anode under different current densities of 100 mAg^−1^, 200 mAg^−1^, 500 mAg^−1^, 1 Ag^−1^, 2 Ag^−1^, and again 100 mAg^−1^, respectively. Similarly, the rate capability of the Si/rGO anode was very poor as compared with the C@Si/rGO anode. The capacity of Si/rGO anode drops sharply with the increase of the testing current density form 100 mAg^−1^ to 500 mAg^−1^. It even drops to almost 0 mAh g^−1^ while the current density is increased to 1 Ag^−1^ and 2 Ag^−1^. The poor rate capability could be explained by the insufficient electron transfer between the Si active material and conductive matrix. When the current density returns to 100 mAg^−1^, the Si/rGO electrode can recover to the specific capacity of 1029/1126 mAh g^−1^. However, as discussed earlier, as the Si/rGO has suffered from continuing a large volume expansion/shrinkage during the discharge/charge process, and therefore, its specific capacity still dramatically drops to almost zero despite the subsequent cycling at the low current density of 100 mAg^−1^. By contrast, the C@Si/rGO anode shows an excellent rate capability. At the high current density of 1 Ag^−1^ and 2 Ag^−1^, it is still capable of delivering the specific capacities of 943/962 mAh g^−1^ and 759/774 mAh g^−1^, respectively. When the current density is switched back to 100 mAg^−1^, the specific capacity of C@Si/rGO film electrode can be recovered to 1117/1126 mAh g^−1^ and is well maintained during following cycling to 100 cycles. The improved cycling stability and rate performance of the C@Si/rGO film electrode compared with the Si/rGO film electrode are explained by the following reasons. Firstly, the self-assembly process between PDDA@Si and GO promote uniform distribution of Si nanoparticle among the GO nanosheets without agglomeration. The self-standing structure has good flexibility to minimize/buffer the volume expansion/shrinkage of Si nanoparticles during the electrochemical cycling. Secondly, the amorphous carbon layer originating from pyrolysis of PDDA and two-dimensional rGO nanosheets jointly guarantee the good connection between Si and conductive framework in entire electrode, which benefits the charge transfer [1,9]. The introduction of the amorphous carbon layer on the surface of Si could suppress the side reaction of the active material and, which could improve the surface/interface stability in the electrolyte. Last but not least, the porous structure formed by cross linking rGO nanosheets also provides a three-dimensional conductive framework and fast lithium ions diffusion channel [11,24]. As a result, taking advantages of dual carbonaceous materials, the C@Si/rGO film anode significantly outperform the Si/rGO film anode.

**Figure 7:**
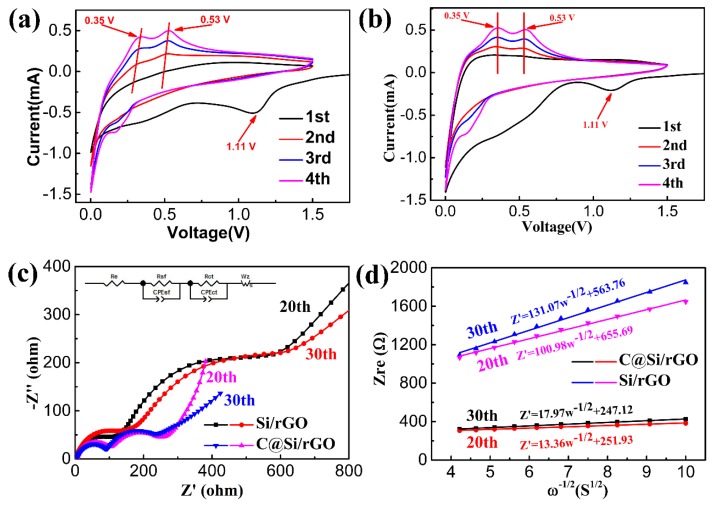
(**a**) Cyclic voltammetry (CV) curves of Si/rGO and (**b**) C@Si/rGO film electrode at a scanning rate of 0.5 mV s^−1^ in the potential range of 0.01–1.5 V (vs. Li/Li^+^); (**c**) Nyquist plots with the equivalent circuit of Si/rGO and C@Si/rGO film electrode after the 20th and 30th cycle; (**d**) Relationships between the real resistance and the frequency of the Si/rGO and C@Si/rGO film electrode after the 20th and 30th cycle. The slope (Warburg impedance coefficient) of which is used to calculate the D_Li_^+^.

Figure 7a,b are the cyclic voltammetry (CV) curves of Si/rGO and C@Si/rGO with a scanning rate of 0.5 mV s^−1^ at the voltage window of 0.01–1.5 V. In the initial cathodic scan, both Si/rGO and C@Si/rGO present a reduction peak at ~1.11 V, and disappear in the following cycles, which are attributed to the formation of SEI film [61,62]. Another main peak between 0.01–0.25 V is ascribed to the Li-alloying reaction [63]. In the anodic scan, two oxidation peaks appear at 0.35 V and 0.53 V, and gradually increase by the following cycle, which are associated with the extraction of lithium ions from Li-Si alloys [64]. It is apparent that the oxidation peaks of Si/rGO electrode move to higher voltages as the cycle proceeds, indicating that the polarization of the electrode increases owing to the poor interfacial stability of the Si/rGO film electrode with an unfavorable SEI layer. However, the positions of oxidation peaks of C@Si/rGO film keep almost unchanged with the cycle increasing, which indicates stable interfacial stability of the C@Si/rGO film electrode with a favorable SEI layer [65].

In order to further confirm the reasons for the excellent performance of the C@Si/rGO anode, the EIS test of the C@Si/rGO and Si/rGO film electrode after 20 and 30 cycles are shown in Figure 7c. Both the C@Si/rGO and Si/rGO film electrodes display two semi-circles in the high frequency region and intermediate frequency region, accompanied with a straight line at low frequency region. The two semi-circles from high to medium frequency represent the surface film resistance and charge transfer resistance of the electrode [66,67]. The straight line in the low frequency region represents the diffusion resistance of the electrode [3,10]. The equivalent circuit for fitting the impedance spectra is displayed as inset Figure 7c, and the corresponding fitting results are shown in Table 2. The R_sf_ of Si/rGO anode after 20 cycles is 92.0 Ω and quickly increases to 116.9 Ω after 30 cycles. However, the R_sf_ of C@Si/rGO anode increases only from 81.04 Ω to 93.60 Ω at the 20th and 30th, which is obviously smaller than that of Si/rGO film electrode, illustrating that more stable interfacial stability for C@Si/rGO film electrode. Furthermore, the R_ct_ of the C@Si/rGO film electrode (113.1 Ω) is much smaller than that of the Si/rGO film electrode (310.6 Ω) at the 20th cycle, which proves the faster charge-transfer reactions for C@Si/rGO film electrode. In addition, the R_ct_ of C@Si/rGO film electrode just deteriorates slightly from 113.1 Ω and 160.2 Ω after 30 cycles, compared with the larger R_ct_ of Si/rGO film electrode (508 Ω), indicating that the electrical connection between active materials and rGO nanosheet could be well maintained during cycling. The results are in good accordance with the better electrochemical performance of the C@Si/rGO film electrode. The result further validates that the dual carbonaceous materials could work together to construct a conductive framework to provide more rapid charge transfer and stable SEI film. The lithium ion diffusion coefficient of the Si/rGO and C@Si/rGO film electrodes at the 20th cycle and 30th could be calculated from the Warburg impedance coefficient (σ_w_) using Equations (3) and (4), [10]

Z_re_ = (R_sf_ +R_ct_ + σ_w_ω^−1/2^) (3)

D_Li_^+^ = R^2^T^2^/(2A^2^n^4^F^4^Cσ_w_^2^) (4)

where, D_Li_^+^ represents the lithium ion diffusion coefficient, *R* is the gas constant (8.314 J·K^−1^·mol^−1^), T is the absolute temperature (298 K), A is area of electrode, which is 1.54 cm^2^ for each piece of electrode, n is the number of electrons transferred, F is Faraday constant, and C is the concentration of lithium ions (1 mol L^−1^). The Warburg impedance coefficient σ_w_ could be determined from the slope of Z_re_ as a function of ω^−1/2^ as shown in Figure 7d. The calculated D_Li_^+^ values for Si/rGO film at the 20th and 30th cycle is 2.29 × 10^−16^ cm^2^ s^−1^ and 1.36 × 10^−16^ cm^2^ s^−1^, respectively. The calculated D_Li_^+^ values for C@Si/rGO film at the 20th and 30th cycle is 7.23 × 10^−14^ cm^2^ s^−1^ and 1.31 × 10^−14^ cm^2^ s^−1^, respectively. The D_Li_^+^ for the C@Si/rGO film is obviously larger than the Si/rGO film, which should be attributed to less agglomeration of Si nanoparticle and the three-dimensional porous structure being favorable to the faster lithium ion transfer.

Figure S3 shows the SEM image of Si/rGO and C@Si/rGO film electrode after 30 cycles in the lithiated state. From Figure 3a, it can be observed that the thickness of the C@Si/rGO film is 64.08 µm, which is approximately 7.26 µm Z-axis expansion compared with the fresh electrode film before cycling (Figure 4a). By contrast, the thickness of Si/rGO film largely increases to 104.54 µm, which is about 87.27 µm Z-axis expansion compared with the fresh electrode film before cycling (Figure 4d). This further indicates that the rGO in the C@Si/rGO film could better buffer the volume expansion of the Si nanoparticle during the lithium ion intercalation process.

Table 3 lists several typical self-supporting composites consisting of Si particle, graphene or other carbon material reported by previous work. It could be observed that the Si/N-doped rGO composite designed in this work presents a rather good electrochemical performance.

## 4. Conclusions

In this paper, a self-standing C@Si/rGO film electrode has been successfully prepared by electrostatic assembly PDDA@Si with graphene oxide followed by freeze drying and carbonization. This hierarchical architecture with Si nanoparticle uniformly distributed among the rGO nanosheet along with a thin carbon layer coating on the surface of Si, synergistically protect the silicon and validate the C@Si/rGO film electrode as a high-performance anode for lithium ion batteries. The C@Si/rGO film electrode display high flexibility and ordered porous structure, which could not only overcome the volume expansion of the Si particle but also provide a fast channel for electron transfer and lithium ion transportation. The dual carbonaceous structure ensures a good connection between Si and the conductive framework, which realizes significant improvement of the electrochemical performance of the silicon-based anode.

## Figures and Tables

**Table 1:** BET and pore data for C@Si/rGO.

Material	S_BET_^[a]^ [m^2^ g^−1^]	S_meso_^[b]^ [m^2^ g^−1^]	V_total_^[c]^ [cm^3^ g^−1^]	V_meso_^[d]^ [cm^3^ g^−1^]	APD ^[e]^
C@Si/rGO	50.0	63.3	0.313	0.315	25.10

^[a]^ Surface areas derived by using the multipoint BET method. ^[b]^ BJH mesopore areas. ^[c]^ Total pore volumes estimated from the amount adsorbed at a relative pressure of P/P0 = 0.99. ^[d]^ Mesopore volume. ^[e]^ Average BJH pore diameters.

**Table 2:** Equivalent circuit parameters obtained from fitting the experimental impedance spectra (Figure 7c).

Sample	R_e_ (Ω)	R_sf_ (Ω)	R_ct_ (Ω)	CPE_1_ (F)	CPE_2_ (F)	W (Ω·s^−1/2^)
Si/rGO	2.37	92.0	310.6	5.34 × 10^−6^	2.52 × 10^−4^	0.42
	2.61	116.9	508	5.82 × 10^−6^	4.10 × 10^−4^	0.44
C@Si/rGO	4.95	81.0	113.1	1.33 × 10^−5^	6.93 × 10^−4^	0.36
	4.29	93.6	160.2	1.65 × 10^−5^	7.26 × 10^−4^	0.60

**Table 3:** Several typical self-supporting composites reported previously consisting of Si particle, graphene or other carbon material.

Samples	Potential Cut-Off (V)	Cycle Number	Initial Columbic Efficiency (CE)	Specific Discharge Capacity (mAh g^−1^)	Current Density (mAg^−1^)	Ref.
C@Si/rGO films	0.01–1.5	100	61.82%	1988	100	This work
Si/rGO film	0.01–3.0	150	53.00%	2030	200	[18]
Si/GP-2	0.02–2.0	100	61.40%	2262	100	[10]
Si/rGO film	0.05–1.2	1300	54.00%	2501	400	[9]
I-rGO/Si	0.00–1.5	100	75.90%	2154	100	[19]
Si-CNT/G paper	0.005–1.5	100	58.00%	2100	200	[20]

**Note:** GP-2 (graphene platelets, Si = 57.6 wt%); I-rGO (iodine-doped graphene); G paper (graphene paper).

## Supplementary Materials

The following are available online at https://www.mdpi.com/2079-4991/9/4/650/s1: Figure S1 (a) SEM image of PDDA@Si, (b-f) EDS mapping of Si, C, O, Cl, N elements on the surface of PDDA@Si. Figure S2 Fourier transform infrared (FT–IR) spectra of KBr, Si and PDDA@Si. Figure S3 SEM images of Si/rGO (a) and C@Si/rGO (b) film electrode after 30 cycles in the lithiated state.

## References

[1] Goodenough J.B., Kim Y. (2010). Challenges for rechargeable Li batteries. Chem. Mater..

[2] Lee W.W., Lee J.M. (2014). Novel synthesis of high performance anode materials for lithium-ion batteries (LIBs). J. Mater. Chem. A.

[3] Yoo H.D., Markevich E., Salitra G., Sharon D., Aurbach D. (2014). On the challenge of developing advanced technologies for electrochemical energy storage and conversion. Mater. Today.

[4] Goodenough J.B. (2018). How we made the Li-ion rechargeable battery. Nature Electron..

[5] Fergus J.W. (2010). Recent developments in cathode materials for lithium ion batteries. J. Power Sources.

[6] Li H., Wang Z., Chen L., Huang X. (2009). Research on advanced materials for Li-ion batteries. Adv. Mater..

[7] Liu N., Wu H., McDowell M.T., Yao Y., Wang C., Cui Y. (2012). A yolk-shell design for stabilized and scalable li-ion battery alloy anodes. Nano Lett..

[8] Wang M.S., Wang Z.Q., Chen Z., Yang Z.L., Tang Z.L., Luo H.Y., Huang Y., Li X., Xu W. (2018). One dimensional and coaxial polyaniline@tin dioxide@multi-wall carbon nanotube as advanced conductive additive free anode for lithium ion battery. Chem. Eng. J..

[9] Zhou X.S., Yin Y.X., Wan L.J., Guo Y.G. (2012). Self-Assembled Nanocomposite of silicon nanoparticles encapsulated in graphene through electrostatic attraction for lithium-ion batteries. Adv. Energy Mater..

[10] Li X., Zhang K., Mitlin D., Yang Z., Wang M., Tang Y., Jiang F., Du Y., Zheng J. (2018). Fundamental insight into Zr modification of Li- and Mn-rich cathodes: combined transmission electron microscopy and electrochemical impedance spectroscopy study. Chem. Mater..

[11] Pan Q., Zuo P., Lou S., Mu T., Du C., Cheng X., Ma Y., Gao Y., Yin G. (2017). Micro-sized spherical silicon@carbon@graphene prepared by spray drying as anode material for lithium-ion batteries. J. Alloys Compd..

[12] Luo Z., Xiao Q., Lei G., Li Z., Tang C. (2016). Si nanoparticles/graphene composite membrane for high performance silicon anode in lithium ion batteries. Carbon.

[13] Fang M., Wang Z., Chen X., Guan S. (2018). Sponge-like reduced graphene oxide/silicon/carbon nanotube composites for lithium ion batteries. Appl. Surf. Sci..

[14] Ru Y., Evans D.G., Zhu H., Yang W. (2014). Facile fabrication of yolk–shell structured porous Si–C microspheres as effective anode materials for Li-ion batteries. RSC Adv..

[15] Yao W., Cui Y., Zhan L., Chen F., Zhang Y., Wang Y., Song Y. (2017). Two-dimensional sandwich-like Ag coated silicon-graphene-silicon nanostructures for superior lithium storage. Appl. Surf. Sci..

[16] Wang G., Xu B., Shi J., Lei X., Ouyang C. (2018). Confined Li ion migration in the silicon-graphene complex system: An ab initio investigation. Appl. Surf. Sci..

[17] Wang M.S., Song W.L., Fan L.Z. (2015). Three-dimensional interconnected network of graphene-wrapped silicon/carbon nanofiber hybrids for binder-free anodes in lithium-ion batteries. ChemElectroChem.

[18] Lu Z., Li B., Yang D., Lv H., Xue M., Zhang C. (2018). A self-assembled silicon/phenolic resin-based carbon core–shell nanocomposite as an anode material for lithium-ion batteries. RSC Adv..

[19] Su M., Wan H., Liu Y., Xiao W., Dou A., Wang Z., Guo H. (2018). Multi-layered carbon coated Si-based composite as anode for lithium-ion batteries. Powder Technol..

[20] Yao W., Chen J., Zhan L., Wang Y., Yang S. (2017). Two-dimensional porous sandwich-like C/Si-graphene-Si/C nanosheets for superior lithium storage. ACS Appl. Mater. Interfaces.

[21] Zhai W., Ai Q., Chen L., Wei S., Li D., Zhang L., Si P., Feng J., Ci L. (2017). Walnut-inspired microsized porous silicon/graphene core–shell composites for high-performance lithium-ion battery anodes. Nano Res..

[22] Li X., Tang Y., Song J., Yang W., Wang M., Zhu C., Zhao W., Zheng J., Lin Y. (2018). Self-supporting activated carbon/carbon nanotube/reduced graphene oxide flexible electrode for high performance supercapacitor. Carbon.

[23] Chen C., Wu M., Wang S., Yang J., Qin J., Peng Z., Feng T., Gong F. (2017). An in situ iodine-doped graphene/silicon composite paper as a highly conductive and self-supporting electrode for lithium-ion batteries. RSC Adv..

[24] Wu P.F., Guo C.Q., Han J.T., Yu K.R., Dong X.C., Yue G.H., Yue H.J., Guan Y., Liu A.H. (2018). Fabrication of double core–shell Si-based anode materials with nanostructure for lithium-ion battery. RSC Adv..

[25] Chen Y.L., Hu Y., Shen Z., Chen R.Z., He X., Zhang X.W., Li Y.Q., Wu K.S. (2017). Hollow core-shell structured silicon@carbon nanoparticles embed in carbon nanofibers as binder-free anodes for lithium-ion batteries. J. Power Sources.

[26] Li Y., Chang B., Li T.T., Kang L.T., Xu S.D., Zhang D., Xie L.L., Liang W. (2016). One-step synthesis of hollow structured Si/C composites based on expandable microspheres as anodes for lithium ion batteries. Electrochem. Commun..

[27] Wang T., Wang F.H., Zhu H. (2015). Hollow core-shell-structured Si-C composites as high-performance anodes for lithium-ion batteries. Mater. Lett..

[28] Wang M.S., Wang Z.Q., Jia R., Yang Z.L., Yang Y., Zhu F.Y., Huang Y., Li X. (2018). Nano tin dioxide anchored onto carbon nanotube/graphene skeleton as anode material with superior lithium-ion storage capability. J. Electroanal. Chem..

[29] Dey A., Bajpai O.P., Sikder A.K., Chattopadhyay S., Khan M.A.S. (2016). Recent advances in CNT/graphene based thermoelectric polymer nanocomposite: A proficient move towards waste energy harvesting. Renew. Sust. Energ. Rev..

[30] Oh S.Y., Oh M.K., Kang T.J. (2013). Characterization and electrorheological response of silica/titania-coated MWNTs synthesized by sol–gel process. Colloid. Surface. A.

[31] Abnavi A., Faramarzi M.S., Abdollahi A., Ramzani R., Ghasemi S., Sanaee Z. (2017). SnO2@a-Si core–shell nanowires on free-standing CNT paper as a thin and flexible Li-ion battery anode with high areal capacity. Nanotechnology.

[32] Suresh S., Wu Z.P., Bartolucci S.F., Basu S., Mukherjee R., Gupta T., Hundekar P., Shi Y., Lu T.M., Koratkar N. (2017). Protecting silicon film anodes in lithium-ion batteries using an atomically thin graphene drape. ACS Nano.

[33] Chang J., Huang X., Zhou G., Cui S., Hallac P.B., Jiang J., Hurley P.T., Chen J. (2014). Multilayered Si nanoparticle/reduced graphene oxide hybrid as a high-performance lithium-ion battery anode. Adv. Mater..

[34] Wang B., Li X., Luo B., Jia Y., Zhi L. (2013). One-dimensional/two-dimensional hybridization for self-supported binder-free silicon-based lithium ion battery anodes. Nanoscale.

[35] Xin X., Zhou X., Wang F., Yao X., Xu X., Zhu Y., Liu Z. (2012). A 3D porous architecture of Si/graphene nanocomposite as high-performance anode materials for Li-ion batteries. J. Mater. Chem..

[36] Mi H., Li F., Xu S., Li Z., Chai X., He C., Li Y., Liu J. (2016). A Tremella-like nanostructure of silicon@void@graphene-like nanosheets cmposite as an anode for lithium-ion batteries. Nanoscale Res. Lett..

[37] Li Y., Yan K., Lee H.-W., Lu Z., Liu N., Cui Y. (2016). Growth of conformal graphene cages on micrometre-sized silicon particles as stable battery anodes. Nat. Energy.

[38] Chen S., Shen L., van Aken P.A., Maier J., Yu Y. (2017). Dual-functionalized double carbon shells coated silicon nanoparticles for high performance lithium-ion batteries. Adv. Mater..

[39] Li P., Zhang K., Park J.H. (2018). Dual or multi carbonaceous coating strategies for next-generation batteries. J. Mater. Chem. A.

[40] Cai H., Han K., Jiang H., Wang J., Liu H. (2017). Self-standing silicon-carbon nanotube/graphene by a scalable in situ approach from low-cost Al-Si alloy powder for lithium ion batteries. J. Phys. Chem. Solids.

[41] Ma X., Hou G., Ai Q., Zhang L., Si P., Feng J., Ci L. (2017). A heart-coronary arteries structure of carbon nanofibers/graphene/silicon composite anode for high performance lithium ion batteries. Sci. Rep..

[42] Tao H., Xiong L., Zhu S., Yang X., Zhang L. (2016). Flexible binder-free reduced graphene oxide wrapped Si/carbon fibers paper anode for high-performance lithium ion batteries. Int. J. Hydrogen Energy.

[43] Li N., Zhou G., Li F., Wen L., Cheng H.-M. (2013). A Self-Standing and Flexible Electrode of Li_4_Ti_5_O_12_ Nanosheets with a N-Doped Carbon Coating for High Rate Lithium Ion Batteries. Adv. Funct. Mater..

[44] Chen Y., Choi S., Su D., Gao X., Wang G. (2018). Self-standing sulfur cathodes enabled by 3D hierarchically porous titanium monoxide-graphene composite film for high-performance lithium-sulfur batteries. Nano Energy.

[45] Choudhury A., Kim J.-H., Yang K.-S., Yang D.-J. (2016). Facile synthesis of self-standing binder-free vanadium pentoxide-carbon nanofiber composites for high-performance supercapacitors. Electrochim. Acta.

[46] Chao Y., Jalili R., Ge Y., Wang C., Zheng T., Shu K., Wallace G.G. (2017). Self-Assembly of Flexible Free-Standing 3D Porous MoS2-Reduced Graphene Oxide Structure for High-Performance Lithium-Ion Batteries. Adv. Funct. Mater..

[47] He J., Li Q., Chen Y., Xu C., Zhou K., Wang X., Zhang W., Li Y. (2017). Self-assembled cauliflower-like FeS2 anchored into graphene foam as free-standing anode for high-performance lithium-ion batteries. Carbon.

[48] Wang M.S., Song W.L., Wang J., Fan L.Z. (2015). Highly uniform silicon nanoparticle/porous carbon nanofiber hybrids towards free-standing high-performance anodes for lithium-ion batteries. Carbon.

[49] Botas C., Carriazo D., Zhang W., Rojo T., Singh G. (2016). Silicon-Reduced Graphene Oxide Self-Standing Composites Suitable as Binder-Free Anodes for Lithium-Ion Batteries. ACS Appl. Mater. Interfaces.

[50] Tang H., Zhang Y.J., Xiong Q.Q., Cheng J.D., Zhang Q., Wang X.L., Gu C.D., Tu J.P. (2015). Self-assembly silicon/porous reduced graphene oxide composite film as a binder-free and flexible anode for lithium-ion batteries. Electrochim. Acta.

[51] Cui L.-F., Hu L., Choi J.W., Cui Y. (2010). Light-Weight Free-Standing Carbon Nanotube-Silicon Films for Anodes of Lithium Ion Batteries. ACS Nano.

[52] Fu K., Yildiz O., Bhanushali H., Wang Y., Stano K., Xue L., Zhang X., Bradford P.D. (2013). Aligned carbon nanotube-silicon sheets: a novel nano-architecture for flexible lithium ion battery electrodes. Adv. Mater..

[53] Liu B., Wang X., Chen H., Wang Z., Chen D., Cheng Y.B., Zhou C., Shen G. (2013). Hierarchical silicon nanowires-carbon textiles matrix as a binder-free anode for high-performance advanced lithium-ion batteries. Sci. Rep..

[54] Zhu J., Yang J., Xu Z., Wang J., Nuli Y., Zhuang X., Feng X. (2017). Silicon anodes protected by a nitrogen-doped porous carbon shell for high-performance lithium-ion batteries. Nanoscale.

[55] Choi H.S., Kim S.J., Choi H.W., Park C.E., Gao Y.J., Hang Y., Jeong S.Y., Kim J.P., B J.S., Cho C.R. (2017). Enhanced cycle stability of silicon nanoparticles coated with nitrogen-doped carbon layer for lithium-ion battery anode. Curr. Appl. Phys..

[56] Yoon T., Cho M., Suh Y.-W., Oh E.-S., Lee J.K. (2011). Reassembled graphene-platelets encapsulated silicon nanoparticles for Li-ion battery anodes. J. Nanosci. Nanotechnol..

[57] Fang S., Tong Z., Nie P., Liu G., Zhang X. (2017). Raspberry-like nanostructured silicon composite anode for high-performance lithium-ion batteries. ACS Appl. Mater. Interfaces.

[58] Li Z.F., Zhang H., Liu Q., Liu Y., Stanciu L., Xie J. (2014). Novel pyrolyzed polyaniline-grafted silicon nanoparticles encapsulated in graphene sheets as Li-ion battery anodes. ACS Appl. Mater. Interfaces.

[59] Chang P., Liu X., Zhao Q., Huang Y., Huang Y., Hu X. (2017). Constructing three-dimensional honeycombed graphene/silicon skeletons for high-performance Li-ion batteries. ACS Appl. Mater. Interfaces.

[60] Liu X.W., Zhong X.W., Yang Z.Z., Pan F.S., Gu L., Yu Y. (2015). Gram-scale synthesis of graphene-mesoporous SnO_2_ composite as anode for lithium-ion batteries. Electrochim. Acta.

[61] Wu X.D., Wang Z.X., Chen L.Q., Huang X.J. (2003). Ag-enhanced SEI formation on Si particles for lithium batteries. Electrochem. Commun..

[62] Xu Y., Yin G., Ma Y., Zuo P., Cheng X. (2010). Simple annealing process for performance improvement of silicon anode based on polyvinylidene fluoride binder. J. Power Sources.

[63] Du C.Y., Gao C.H., Yin G.P., Chen M., Wang L. (2011). Facile fabrication of a nanoporous silicon electrode with superior stability for lithium ion batteries. Energy Environ. Sci..

[64] Jiang T., Zhang S., Qiu X., Zhu W., Chen L. (2007). Preparation and characterization of silicon-based three-dimensional cellular anode for lithium ion battery. Electrochem. Commun..

[65] Su J.M., Zhang C.C., Chen X., Liu S., Huang T., Yu A.S. (2018). Carbon-shell-constrained silicon cluster derived from Al-Si alloy as long-cycling life lithium ion batteries anode. J. Power Sources.

[66] Yariv O., Hirshberg D., Zinigrad E., Meitav A., Aurbach D., Jiang M., Powell B.R. (2014). Carbon negative eectrodes for Li-ion batteries: the effect of solutions and temperatures. J. Electrochem. Soc..

[67] Li X., Zhang K., Wang M., Liu Y., Qu M., Zhao W., Zheng J. (2018). Dual functions of zirconium modification on improving the electrochemical performance of Ni-rich LiNi_0.8_Co_0.1_Mn_0.1_O_2_. Sustain. Energy Fuels.

