# Supplementary material for: Dual Carbonaceous Materials Synergetic Protection Silicon as a High-Performance Free-Standing Anode for Lithium-Ion Battery
Source: Nanomaterials (Basel). 2019 Apr 23;9(4):650. doi: 10.3390/nano9040650 (PMC6523080; doi:10.3390/nano9040650)
Supplement: Supplementary file 1 [file nanomaterials-09-00650-s001.pdf]

Article

Dual Carbonaceous Materials Synergetic Protection Silicon as a High-Performance Free-Standing Anode for Lithium-Ion Battery

Xing Li [1,*], Yongshun Bai [1], Mingshan Wang [1,*], Guoliang Wang [1], Yan Ma [1], Yun Huang [1] and Jianming Zheng [2,*]

[1] The Center of New Energy Materials and Technology, School of Materials Science and Engineering, Southwest Petroleum University, Chengdu 610500, Sichuan, China; bysdyr@163.com (Y.B.); wangguoliang1012@163.com (G.W.); mayanlouis@163.com (Y.M.); huangyun982@163.com (Y.H.)

[2] Research Institute (RI), NingDe Amperex Technology Limited, Ningde 352100, Fujian, China

* Correspondence: lixing198141@yahoo.com (X.L.); ustbwangmingshan@163.com (M.W.); xmzhjm@126.com (J.Z.)

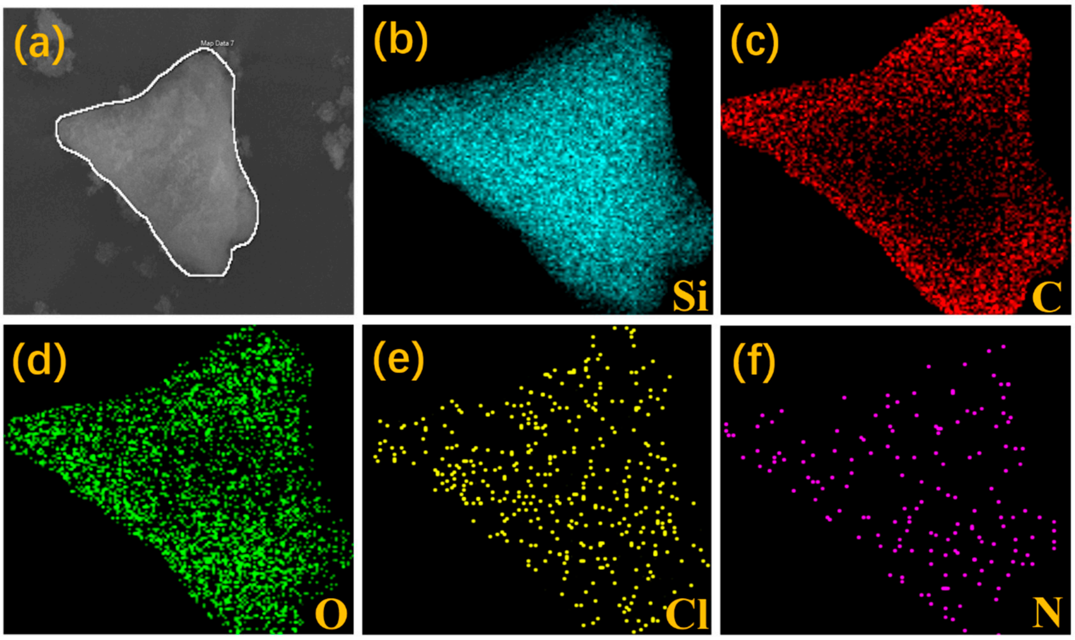

Figure S1 (a) The SEM image of PDDA@Si, (b-f) The EDS mapping of Si, C, O, Cl, N elements on the surface of PDDA@Si.

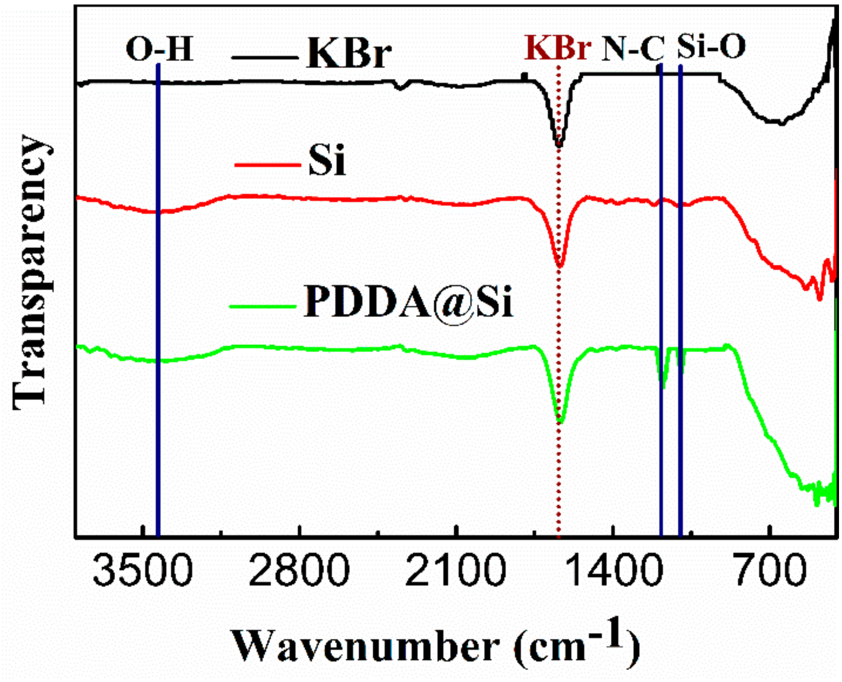

Figure S2 Fourier transform infrared (FTIR) spectra of KBr, Si and PDDA@Si.

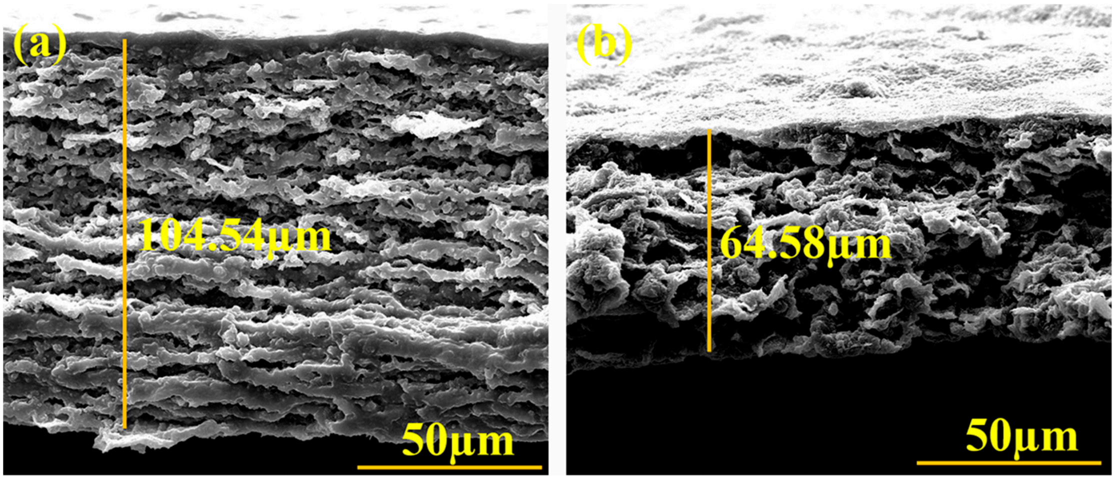

Figure S3 SEM images of Si/rGO (a) and C@Si/rGO (b) film electrode after 30 cycles in the lithiated state.
